# Report of the Salaries Committee of the College of Nursing

**Published:** 1919-06-28

**Authors:** 


### SALARIES AND CONDITIONS OF EMPLOYMENT OF NURSES.

### REPORT OF THE SALARIES COMMITTEE OF THE COLLEGE OF NURSING.

(Concluded from page 278).

The Report of the Salaries and Conditions of Employment Committee of the College of Nursing is probably the most important single document affecting nurses which has issued from the Press since Florence Nightingale began her noble work. This is so because from its nature it must become historical, and endure as an authoritative statement and basis, which is likely to be referred to times out of mind as the world progresses and endures. This being so, every trained nurse who loves her profession, honours it, and is resolved to do her part to raise and develop it to the utmost, should obtain a copy and preserve it amongst the treasures which have come to her in the course of her professional career. We have thought it just and helpful to make this declaration as a foreword to the concluding portion of this Report.

A copy of the Report has now been circulated to institutions and to those who have supplied statistics to the committee. The Council of the College has the Report under consideration, and in due course will formulate suggestions based upon it. Copies of the Report may be obtained from the publishers, Messrs. Eyre & Spottiswoode, Ltd., East Harding Street, London, E.C. 4, or from **The Scientific Press, Limited, 28 and 29 Southampton Street, London, W.C.2, price 6d., or by post 7d.**

### The 48-Hour Week: Illustrations of its Working.

The following time-tables, taken at random from the returns, have been adjusted to give an average duty week of 48 hours, and serve to show that whilst hospitals may find overlapping periods of seven hours a day (excluding meals) unworkable, the average working week of 48 hours is both possible and practicable. The overlapping periods referred to are:—

12 midnight to 8.30 A.M., with $1\frac{1}{2}$ hour for meals.
8 A.M. to 5 P.M., with 2 hours for meals.
4.30 P.M. to 12.30 A.M., with 1 hour for meals.

### ILLUSTRATION I. (a)—DAY WORK.

TIME-TABLE FOR WARD SISTERS AND ASSISTANT NURSES.

Wards, 8 A.M. Lunch, 9.30-10. Dinner, 1-2. Tea, 4 or 4.30. Supper, 8.30 P.M.

*Leave of Absence.*—Daily: Two hours. Weekly: Half-day from 1 to 10 P.M. Monthly: One day. Sundays: Off duty till 12 noon, or 4.30 to 10 P.M.

Assuming that the nurses work from 8 A.M. to 8.30 P.M., less two hours for meals taken outside the wards, then the working day is $10\frac{1}{2}$ hours, or $73\frac{1}{2}$ hours per week.

| | |
|---|---|
| Daily off duty of two hours for five days is | 10 hours. |
| Weekly half-day from 1 to 10 P.M. is ... ... | 6 ,, |
| Monthly one day, taken as half-day extra on one week in four, is 8 A.M. to 1 P.M., less half-hour for lunch—*i.e.*, $4\frac{1}{2}$ hours, or a weekly average of ... ... ... ... | $1\frac{1}{8}$ ,, |
| Sundays in both cases is ... ... ... ... | $3\frac{1}{2}$ ,, |
| | $20\frac{5}{8}$ ,, |

Seventy-three and a-half hours, less $20\frac{5}{8}$ hours, is $52\frac{7}{8}$ hours, which is $4\frac{5}{8}$ hours in excess of the 48 advised. In order to reduce this time, allow three hours off daily on five days instead of two hours, or alternately:

| | |
|---|---|
| Two hours off on four days ... ... ... | 8 hours. |
| Three and a-half hours on Sundays and one week-day ... ... ... ... ... ... | 7 ,, |
| One whole day in seven ... ... ... ... | $10\frac{1}{2}$ ,, |
| | $25\frac{1}{2}$ ,, |

Seventy-three and a-half hours less $25\frac{1}{2}$ hours is 48 hours.

(*b*) PROBATIONERS IN THE SAME INSTITUTION.

Wards, 7.15 A.M.; lunch, 9—9.30; dinner, 12—1; tea, 4—4.30; supper, 8.15 P.M.

*Leave of Absence.*—Weekly, half-day from 2 to 10 P.M.; daily, two hours; monthly, one day; Sundays, alternately from 10 to 1 P.M., or from 4.30 to 10 P.M.

The probationers have a working day of 11 hours, or 77 hours a week.

The off-duty times work out at $19\frac{5}{8}$ hours a week:

| | |
|---|---|
| One half-day a week for three weeks ... | $5\frac{3}{4}$ hours. |
| Daily two hours for five days ... ... ... | 10 ,, |
| One day on the fourth week in the month | $1\frac{1}{4}$ ,, (approx.) |
| Sundays, 10 to 1 P.M., 2 hours; *or* 4.30 to 10 P.M., $3\frac{1}{4}$ hours ... Average | $2\frac{5}{8}$ ,, |
| | $19\frac{5}{8}$ ,, |

This is approximately $57\frac{1}{2}$ hours on duty a week.

These hours may be reduced to 48 by:

| | |
|---|---|
| Three hours on five days ... ... ... | 15 hours. |
| Half-day weekly ... ... ... ... ... | $5\frac{3}{4}$ ,, |
| A day a fortnight in addition to the weekly half-day ... ... ... ... ... ... | 4 ,, |
| Sundays, 8.45 A.M. to 1 P.M., $2\frac{3}{4}$ hours; or 1 P.M. to 10 P.M., $5\frac{3}{4}$ hours ... ... ... | $4\frac{1}{4}$ ,, |
| | 29 ,, |

Seventy-seven hours less 29 hours is 48 hours; *or*

| | |
|---|---|
| Three hours on five days ... ... ... | 15 hours. |
| One day a week ... ... ... ... ... | 11 ,, |
| Sundays, 10 to 1 P.M., or 3.45 to 10 P.M. ... | 3 ,, |
| | 29 ,, |

Seventy-seven hours less 29 hours is 48 hours.

(*c*) NIGHT DUTY IN THE SAME INSTITUTION.

In the wards 8.15 P.M. to 8.30 A.M., less one hour during the night for meals, makes a night of $11\frac{1}{4}$ hours and a week of $78\frac{3}{4}$ hours.

The leave of absence given is:

| | |
|---|---|
| Alternate Sundays to 10 P.M., three nights off a fortnight, together giving weekly ... | $17\frac{3}{4}$ hours. |
| One evening weekly until 10 P.M. ... ... | $1\frac{3}{4}$ ,, |
| | $19\frac{1}{2}$ ,, |

This gives $59\frac{1}{4}$ hours a week. To reduce to 48 the institution may allow five nights off a fortnight, instead of three, which is not to be recommended or, as there is at present $1\frac{1}{4}$ hours when all the probationers are in the wards together, shorten the night nurses' hours from 8.15 P.M. to 7.30 A.M. This makes a working week of $71\frac{3}{4}$ hours. If $1\frac{3}{4}$ hours were allowed for a meal and rest between the hours of 12 and 4 A.M., then the working hours

**Salaries and Conditions of Employment—(*cont.*)**

would be reduced to 66½ hours. The same hours of off duty now work out at:

| | |
|---|---|
| Alternate Sundays until 10 P.M. ... ... | ⅞ hours. |
| Three nights a fortnight ... ... ... | 14¼ ,, |
| One evening weekly until 10 P.M. ... ... | 1¾ ,, |
| | 16⅞ ,, |

The remaining 1⅝ hours being allowed to accumulate for twelve weeks amount to 19½ hours, and can be given as two nights off duty, or one night and one day before going on day duty.

### ILLUSTRATION II.—NIGHT DUTY.

The following illustration of night duty works out more clearly:

| | |
|---|---|
| Night nurses on duty 8.20 P.M. to 8.20 A.M. | 84 hours. |
| Allow one hour for meals and one hour for rest and one night off weekly ... ... | 60 ,, |

If an additional week-end of two nights a fortnight and one week night off duty to 10.20 P.M. be allowed, the hours reduce to 48 a week.

### ILLUSTRATION III. (a)—DAY DUTY.

The hospital from which the following time-tables are taken allows one day off in seven to nurses on day duty.

*Probationers.*

On duty 7 A.M. to 9 P.M.

Allowing two hours off for meals, the duty hours amount to 72 a week.

Off duty, 9 A.M. to 1 P.M.—2½ hours; *or* 1 P.M. to 5 P.M.—3½ hours; *or* 6 P.M. to 10 P.M.—3 hours (if the midday meal be at 12 noon).

This reduces the duty hours by 18, *i.e.*, to 54 hours a week. In order to reduce this to 48 hours a week, change one of the 6 P.M. to 10 P.M. days to 1 P.M. to 10 P.M., and allow a week-end from Saturday at 9 A.M. to Monday at 9 A.M. every three months.

### (b).—NIGHT DUTY.

9 P.M. to 7.30 A.M., less one hour allowed for meals between 12 and 3 A.M., is 66½ hours a week. To reduce to 48 hours allow an extra hour for rest in the night, a night a week, and two extra nights every two months.

In order to reduce the hours of duty to an average of 48 hours a week, without allowing nights off duty during the period for which the nurse is on night duty, it will be necessary to shorten the night nurses' hours by the nurse going on duty at a later hour. If the night nurses go on duty from 10 P.M. to 7 A.M., with two hours out of the wards during the night, the weekly hours on duty amount to 49. At the end of three months an excess of 12 hours will have accumulated. As many hospitals allow at least two nights off duty before going on day duty, this considerably lessens the average weekly hours.

If the hours of night duty are 9.30 P.M. to 8 A.M., and two hours are allowed off duty nightly, then the nurse works 59½ hours a week. It is, therefore, evident that the accumulation of off-duty hours will be considerable at the end of three months (138 hours, or 16 nights of 8½ hours and 4 hours extra).

An easy method would be to fix the night hours to give an average of 48 hours, and then to work two overlapping periods of day duty, thus:—

*Night Duty.*—10 P.M. to 7 A.M., two hours out of the wards nightly, and two nights off every three months.

*Corresponding Day Duty.*—6 A.M. to 6 P.M., less four hours for meals and off duty, and one day in seven, the off-duty hours being given in the morning, and 9.30 A.M. to 1 P.M., with 5 P.M. to 10.30 P.M. on six days a week.

*These examples are given solely as illustrations.* The method of adjusting the time-tables must of necessity vary with the nursing institution.

### General Conditions.

### OFF DUTY.

A nurse writes:

"When nurses live any distance from the hospital, so much time is spent in getting on and off duty that one's two hours off is often reduced to one and a-quarter hours."

From the evidence before the Committee it is clear that off-duty time is totally inadequate. Wherever possible it is advised that as long a period of free time be given as can be arranged. Hospitals are often situated in the most populated districts of the towns, and the off-duty time daily should be sufficient to allow the nurse to take a 'bus or tram out and back, leaving one hour at least for recreation. Moreover, the rest to nurses is considerably greater if days and half-days are allowed more frequently, and the nurse will lose the "barred in" feeling which often at present exists. One day off in seven is advised. One hospital allows a day a week, and a half-day in addition once a fortnight.

Many institutions allow one night off in seven when nurses are on night duty. Exception is taken to this as being detrimental to the sleep of the nurses on resuming duties, and it is felt that nurses would appreciate the shortening of the night hours by going on duty later and coming off duty earlier, where this is possible. The night hours can also be considerably shortened by allowing an hour to two hours out of the wards between the hours of 12 and 4 A.M.

### Accommodation.

Many letters have been received on the subject of accommodation.

The following letter was received from a nurse who had been in a private nursing home in the West End of London:

"For two months I was working at a nursing home. . . . The salary of trained nurses was £40 per annum, board, lodging, and washing, no uniform allowance. I was promised comfortable quarters. The sleeping quarters assigned me were with two other nurses in a converted mews, the mews on either side being retained for their original purpose, the converted stalls being used as cubicles.

"There was no sitting-room for the nurses' use, and tea was in what was known as the nurses' kitchen, a passage room from one house to the other. All the trays were carried by the nursing staff from the ground floor to the patient's room. The nursing staff had also to assist in stretchering the operation cases from the theatre to their rooms. The domestic arrangements were chaotic.

"Yours faithfully,

————."

On this subject also a Matron writes as follows:

"In most hospitals it is the rule that of the male sex only fathers and brothers may be entertained by the nurses; on no account may a student call, and it is rarely rendered possible for a male friend to be entertained—that a man should call in the evening is almost unheard of. Many of the nurses live far away from home, and if they cannot entertain their friends in the hospital they are forced to meet them in the street. On this last point there will be the greatest opposition, but I can only say that the Matron should know her staff a great deal better than she does."

The Committee is of opinion that the following general accommodation should be allowed: (1) Dining-room; (2) sitting-room; (3) bathrooms, at least one to twelve nurses; (4) adequate lavatory accommodation; (5) where possible, a rest and silence room and a visitors' room.

Each nurse, whether in an institution, home for nurses, or working in a private nursing home, should have a separate bedroom, and it is recommended that all nursing institutions, including private nursing homes, should be subject to inspection at irregular intervals by competent women not attached to the staff of any nursing institution.

The Committee recommends the establishment of separate nurses' homes.

(*Continued on p. iv.*)

